# Kidney transplantation outcomes in children with WT1-associated kidney disease: a single-center cohort study

**DOI:** 10.3389/fped.2026.1884378

**Published:** 2026-07-24

**Authors:** Reyila Abasi, Lin Zhilang, Jiang Mengjie, Liu Longshan, Jiang Xiaoyun, Pei Yuxin

**Affiliations:** 1 Department of Pediatric Nephrology and Rheumatology, The First Affiliated Hospital of Sun Yat-sen University, Guangzhou, China; 2 Organ Transplant Center, The First Affiliated Hospital, Sun Yat-sen University, Guangzhou, Guangdong, China

**Keywords:** children, kidney transplantation outcomes, monogenic kidney disease, pediatric kidney transplantation, WT1 gene

## Abstract

**Introduction:**

WT1-associated kidney disease is an important cause of early-onset end-stage kidney disease (ESKD) in children and may be accompanied by Wilms tumor, gonadal tumors, and disorders of sex development (DSD), creating distinctive challenges for kidney transplantation and post-transplant management.

**Methods:**

We retrospectively analyzed 13 children with WT1-associated kidney disease who underwent kidney transplantation at a single center between February 2020 and October 2025. Clinical characteristics, genetic findings, transplant outcomes, and post-transplant complications were reviewed.

**Results:**

The median age at disease onset was 2.3 years, the median age at progression to ESKD was 4.1 years, and the median age at transplantation was 5.3 years. Three patients developed Wilms tumor, and three phenotypic females had a 46,XY karyotype with gonadal dysgenesis. Twelve distinct WT1 variants were identified, predominantly exon 9 missense variants and intron 9–10 splice-site variants. After a median follow-up of 32 months, all grafts remained functional, with a median estimated glomerular filtration rate of 68.7 mL/min/1.73 m². Epstein–Barr virus viremia occurred in five patients, and two developed post-transplant lymphoproliferative disorder. No disease recurrence was observed.

**Discussion:**

Kidney transplantation achieved favorable short- to mid-term graft outcomes in this cohort. Multidisciplinary surveillance of tumor history, gonadal abnormalities, and post-transplant viral complications remains essential.

## Introduction

Pathogenic variants in WT1 are responsible for a spectrum of developmental disorders affecting the kidney, gonads, and tumor susceptibility, collectively referred to as WT1-related disorders (1). Clinical manifestations include steroid-resistant nephrotic syndrome, diffuse mesangial sclerosis, focal segmental glomerulosclerosis, Denys–Drash syndrome, Frasier syndrome, disorders of sex development (DSD), and increased risk of Wilms tumor (2, 3). These manifestations reflect the important role of WT1 in urogenital development and tumor suppression.

Renal involvement is a nearly universal feature of WT1-associated disease. Many affected children experience rapid progression to early-onset chronic kidney disease (CKD) or end-stage kidney disease (ESKD), making kidney transplantation the definitive form of renal replacement therapy in this population (4). Previous reports have generally demonstrated favorable graft outcomes with minimal risk of disease recurrence following transplantation (5). However, WT1-associated disease is characterized by multisystem involvement, including Wilms tumor, gonadal abnormalities and disorders of sex development, which may pose unique challenges for kidney transplantation management.

Data on kidney transplantation in children with WT1-associated kidney disease remain limited. Existing studies have largely focused on natural history and genotype–phenotype correlations, with limited attention to transplantation-specific management strategies integrating renal, oncologic, and gonadal phenotypes (6).

In this study, we present a single-center cohort of children with WT1-associated kidney disease who progressed to ESKD and underwent kidney transplantation. We aimed to characterize pre-transplant renal, oncologic, and DSD-related phenotypes, describe short- to mid-term transplant outcomes, and explore their implications for clinical management.

## Methods

This retrospective study included children with WT1-associated kidney disease who progressed to ESKD and underwent kidney transplantation at the First Affiliated Hospital of Sun Yat-sen University between February 2020 and October 2025. Thirteen patients met the inclusion criteria. Clinical records were reviewed to obtain demographic data, age at onset, initial presentation, kidney biopsy findings, and time from onset to ESKD. Tumor-related and DSD-related information, including imaging findings, surgical reports, endocrine assessments, and oncologic treatment history, were collected when applicable.

Genetic data were reviewed for all children. WT1 variants identified by whole-exome sequencing were confirmed by Sanger sequencing when necessary, and variant pathogenicity was classified according to American College of Medical Genetics and Genomics (ACMG) guidelines (7). Transplant-related information included age at transplantation, donor type, induction and maintenance immunosuppression, interval from ESKD to transplantation, and rejection episodes defined according to Banff 2019 criteria (8). Serial graft function, viral monitoring for Epstein–Barr Virus (EBV), BK polyomavirus, Cytomegalovirus (CMV), and JC polyomavirus were recorded throughout follow-up, together with surgical and vascular complications.

Estimated glomerular filtration rate (eGFR) was calculated using the updated Schwartz formula (9). ESKD was defined as eGFR <15 mL/min/1.73 m^2^ or dialysis dependence (10); graft failure was defined as return to dialysis or need for re-transplantation (11). Post-transplant lymphoproliferative disorder (PTLD) was diagnosed based on histopathological evaluation of lymph node or extranodal tissue (12). Statistical analyses were performed using SPSS version 25.0. Continuous variables were reported as mean ± SD or median with interquartile ranges, and categorical variables as counts and percentages. Given the small sample size, analyses were primarily descriptive.

The study was approved by the Ethics Committee of the First Affiliated Hospital of Sun Yat-sen University (Approval No. [2021]137-1), with waiver of informed consent due to its retrospective design.

## Results

### Baseline clinical characteristics

Thirteen children with WT1-associated kidney disease were included. The median age at disease onset was 2.3 years (range 0.5–7.0 years), and the median age at progression to ESKD was 4.1 years (range 1.5–13.8 years). The interval from onset to ESKD ranged from 0 to 152 months. The cohort included nine female presenting and four male presenting, three female presenting were found to have a 46,XY karyotype.

Initial presentations included proteinuria in nine children (69.2%) and kidney dysfunction in four (30.7%). Three children presented with an abdominal mass and were subsequently diagnosed with Wilms tumor. Six children underwent kidney biopsy, revealing focal segmental glomerulosclerosis (*n* = 3), crescentic glomerulonephritis (*n* = 1), atypical membranous nephropathy (*n* = 1), and proliferative–sclerotic glomerulonephritis (*n* = 1). Baseline characteristics are summarized in Table 1.

**Table 1:** Baseline characteristics of 13 children with germline WT1 variants.

Characteristic	Number of patients
Sex	13
Male presenting	4
Female presenting	9
46,XY female phenotype	3
Age at onset, years	2.3 (range 0.5–7.0)
Kidney presentation	
Proteinuria	9
Kidney dysfunction	4
Kidney Biopsy (*n* = 6)	FSGS (3), crescentic GN (1), atypical MN (1), proliferative–sclerotic GN (1)
Wilms tumor	3 (2 bilateral)
Time from onset to ESKD, months	24 (range 0–152)
Age at ESKD, years	4.1 (range 1.5–13.8) [IQR, 2.5–10.6]

IQR, interquartile range; FSGS, focal segmental glomerulosclerosis; GN, glomerulonephritis; MN, membranous nephropathy; ESKD, end-stage kidney disease.

### Tumor-related phenotype

Three children were diagnosed with Wilms tumor, all of whom initially presented with an abdominal mass. One child was diagnosed with a right-sided Wilms tumor at 1 year of age, underwent unilateral nephrectomy and chemotherapy, and progressed to ESKD at 13 years 9 months before receiving transplantation at 14 years. The second child had bilateral Wilms tumors diagnosed in infancy, underwent bilateral nephron-sparing surgery at 1 year of age, developed ESKD at 6 years 11 months, and subsequently underwent bilateral nephrectomy at the time of kidney transplantation at 8 years 10 months. The third child was diagnosed with bilateral Wilms tumors in early childhood, underwent bilateral nephron-sparing surgery at 1 year of age, progressed rapidly to ESKD at 2 years 5 months, and underwent bilateral nephrectomy and kidney transplantation during the same operation at 3 years 4 months. The interval between completion of tumor therapy and kidney transplantation ranged from 28 to 156 months. No post-transplant Wilms tumor recurrence was observed.

### DSD-related phenotype

Three presenting females carried a 46,XY karyotype. Two carried intron 9 splice-site variants and one carried an exon 9 missense variant. All displayed gonadal dysgenesis. One child had ovotesticular DSD and underwent bilateral gonadectomy at the time of kidney transplantation at 12 years. A second child had asymmetric gonadal dysgenesis with a confirmed gonadoblastoma and underwent bilateral gonadectomy prior to transplantation, followed by estrogen replacement therapy. The third child underwent prophylactic gonadectomy at an outside institution before referral; detailed operative information was unavailable. No additional gonadal tumors were detected during follow-up.

### Genetic findings

Twelve distinct WT1 variants were detected among the thirteen children, including two previously unreported missense variants (c.1405G>A and c.1333C>T). Exon 9 variants were the most frequent (8/13), followed by intron 9–10 splice-site variants (3/13), with additional variants in exons 8 and 4. Variant characteristics are summarized in Table 2.

**Table 2:** WT1 variant summary.

Case	Variant (cDNA)	Protein change	Type	Region	Chromosome karyotype	Gonadal findings	Wilms tumor	Kidney phenotype
P1	c.1405G>A	p.D469N	Missense	Exon 9	46 XX	Normal	No	Proteinuria, Kidney dysfunction
P2	c.1432+1G>T	NA	Splice	Intron 9	46 XY	46,XY female phenotype	No	Proteinuria
P3	c.1447+4C>T	NA	Splice	Intron 9	46 XX	Normal	No	Proteinuria
P4	c.1393C>T	p.H465Y	Missense	Exon 9	Not available	Cryptorchidism	No	Proteinuria
P5	c.1432+5G>A	NA	Splice	Intron 9	46 XY	46,XY female phenotype, Gonadoblastoma	No	Proteinuria
P6	c.1399C>T	p.R467W	Missense	Exon 9	46 XX	Normal	No	Kidney dysfunction
P7	c.1405G>A	p.D469N	Missense	Exon 9	Not available	Cryptorchidism	No	Proteinuria, Kidney dysfunction
P8	c.1387C>T	p.R463X	Nonsense	Exon 9	Not available	Normal	Yes	
P9	c.754G>A	p.D252N	Missense	Exon 9	46 XY	46,XY female phenotype	No	Proteinuria, Kidney dysfunction
P10	c.260C>T	p.S87F	Missense	Exon 4	46 XX	Normal	No	Proteinuria
P11	c.736C>T	p.R246X	Nonsense	Exon 9	Not available	Normal	Yes	
P12	c.1333C>T	p.H445Y	Missense	Exon 8	Not available	Normal	Yes	Proteinuria
P13	c.748C>T	p.R250W	Missense	Exon 9	Not available	Cryptorchidism, Hypospadias	No	

CKD, chronic kidney disease; NA, not applicable.

### Transplant characteristics and outcomes

All children received allograft kidney transplantation at a median age of 5 years 4 months (range 3–14 years). The interval from ESKD to transplantation was 11 months (range 1–42 months). All recipients received calcineurin inhibitor–based maintenance immunosuppression with mycophenolate mofetil (or mycophenolate sodium).

At a median follow-up of 32 months (range 2–70 months), all patients were alive with functioning grafts. The median eGFR at last follow-up was 68.7 mL/min/1.73 m^2^ (IQR 58.1–82.4 mL/min/1.73 m^2^). No cases of recurrent proteinuria were observed. No acute rejection occurred during the first 6 months after transplantation. Three children developed chronic antibody-mediated rejection or mixed rejection at 8, 27, and 67 months after transplantation, respectively; however, all maintained stable graft function without requiring dialysis.

**Figure 1:**
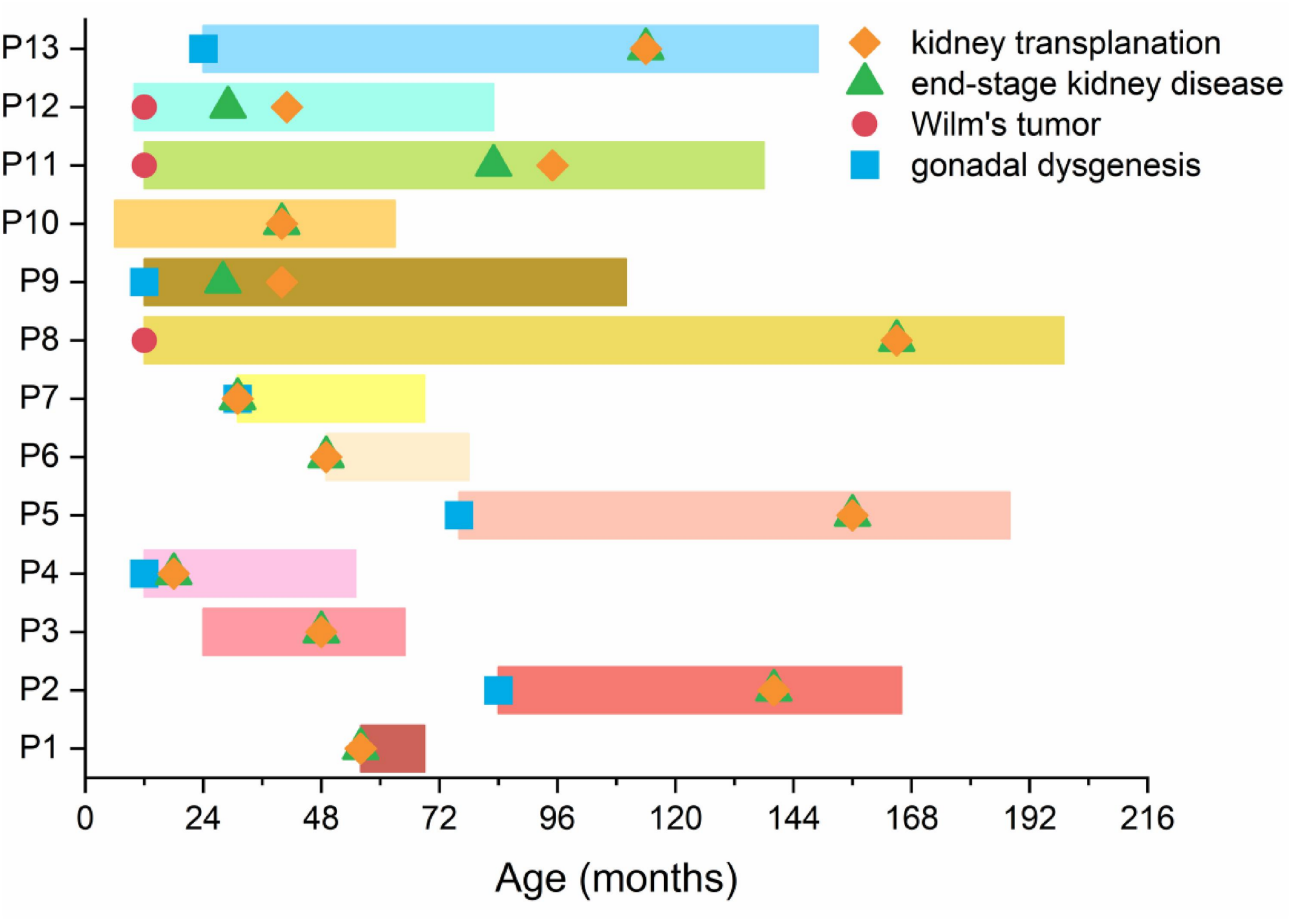
The clinical timeline of disease progression in children with WT1-associated kidney disease.

EBV viremia occurred in five children. Two developed generalized lymphadenopathy at 3 and 9 months post-transplant, respectively, and were diagnosed with post-transplant lymphoproliferative disorder (PTLD) on biopsy. In both cases, tacrolimus was replaced with cyclosporine, while mycophenolate mofetil was continued as part of the maintenance immunosuppression regimen. In one child, peripheral blood EBV DNA became undetectable, and tacrolimus was reintroduced; EBV remained undetectable during two years of follow-up. Another child developed airway obstruction and otitis media and showed clinical improvement, with clearance of EBV viremia, after rituximab therapy. Other viral complications included JC polyomavirus infection in one child at 2 years 8 months post-transplant, BK polyomaviruria in one child at 4 months post-transplant, and CMV pneumonia in one child; all resolved with appropriate management. Non-viral complications included renal artery stenosis in two children, both treated successfully with percutaneous angioplasty. No urinary tract obstruction, vascular thrombosis, recurrent glomerulopathy, or *de novo* malignancy were identified. A timeline summarizing disease progression, treatment, and transplantation is presented in Figure 1.

## Discussion

WT1-associated kidney disease represents a complex, multisystem disorder in which renal, gonadal, and oncologic manifestations unfold across childhood (13). In this cohort, kidney transplantation was associated with excellent short- to mid-term outcomes, and no child experienced graft loss or disease recurrence. These findings are consistent with prior reports indicating that WT1-associated kidney disease does not recur in the allograft and that transplantation is an effective long-term treatment strategy (5).

Interestingly, none of the children in our cohort had diffuse mesangial sclerosis (DMS), a classic pathological feature of early-onset WT1 nephropathy, particularly Denys–Drash syndrome. Several factors may account for this finding. Most patients in our cohort carried exon 9 missense variants or intron 9–10 splice-site variants, which are more frequently associated with focal segmental glomerulosclerosis or later-onset nephropathy than with infantile DMS. In addition, native kidney biopsy was performed in only six patients, limiting histopathological assessment in the remainder of the cohort. Therefore, our findings should be interpreted in the context of the genetic characteristics of our patients and the limited availability of native kidney pathology.

A distinctive feature of WT1-associated kidney disease is the complexity of pre-transplant management. In our cohort, three children presented with Wilms tumor as the initial manifestation, highlighting the importance of recognizing underlying WT1-associated disease. The interval between completion of tumor therapy and kidney transplantation ranged from 28 to 156 months, and no post-transplant tumor recurrence was observed. Although our data do not define a minimum safe waiting period, these findings support individualized transplant planning after adequate oncologic control. More aggressive prophylactic strategies have also been reported in children with WT1 variants. A case from Japan described a child with Denys–Drash syndrome who underwent prophylactic bilateral nephrectomy followed by kidney transplantation prior to the development of Wilms tumor or end-stage kidney disease, aiming to eliminate tumor risk in the context of a WT1 mutation (14). Although such an approach remains controversial, it highlights the broad spectrum of pre-transplant decision-making in WT1-associated kidney disease and underscores the need for individualized decisions regarding nephrectomy timing and transplantation, balancing oncologic risk against preservation of renal function (15).

Gonadal involvement represents another important aspect of WT1-associated kidney disease (16, 17). A nationwide cohort from the Netherlands reported a higher prevalence of urogenital malformations/disorders of sex development in patients with a 46,XY karyotype (18). In our cohort, all three patients with a 46,XY karyotype had gonadal dysgenesis, and two underwent gonadectomy, including one with gonadoblastoma. No additional gonadal tumors were identified during follow-up. These observations are consistent with Drayer et al., which highlighted the heterogeneity of gonadal phenotypes and the need for individualized long-term management in children with WT1 variants (15). Decisions regarding prophylactic gonadectomy should therefore be individualized and made jointly with patients and their families, balancing tumor risk against preservation of gonadal function.

An unexpected observation was the frequency of viral complications, particularly EBV viremia and PTLD. Two children developed PTLD within the first post-transplant year, both requiring treatment. While conclusions are limited by sample size, this finding suggests that children with WT1-associated disease may benefit from intensified EBV monitoring and cautious immunosuppression adjustment. Whether this susceptibility reflects younger transplant age, immunologic characteristics of WT1-associated kidney disease, or cumulative immunosuppression remains to be clarified in larger studies. Most of the post-transplant complications observed in our cohort are recognized complications of kidney transplantation and immunosuppression, rather than disease-specific manifestations.

Taken together, the clinical course of WT1-associated disease is best understood as a continuum in which renal decline, tumor predisposition, gonadal dysgenesis, and post-transplant immune vulnerability interact over time. Successful kidney transplantation represents an important milestone rather than the endpoint of care, and long-term multidisciplinary surveillance remains essential for the management of WT1-associated disease.

## Conclusion

In this single-center cohort, children with WT1-associated kidney disease demonstrated excellent short- to mid-term graft and patient survival following kidney transplantation. However, WT1-associated kidney disease is distinguished by tumor- and DSD-associated risks and an appreciable burden of viral complications, including PTLD. An individualized management approach, incorporating tumor surveillance, gonadal evaluation, and post-transplant monitoring, may help optimize long-term outcomes.

## Data Availability

The datasets presented in this study can be found in online repositories. The names of the repository/repositories and accession number(s) can be found in the article/Supplementary Material.
